# PARP10 Mediates Mono-ADP-Ribosylation of Aurora-A Regulating G2/M Transition of the Cell Cycle

**DOI:** 10.3390/cancers14215210

**Published:** 2022-10-24

**Authors:** Simone Di Paola, Maria Matarese, Maria Luisa Barretta, Nina Dathan, Antonino Colanzi, Daniela Corda, Giovanna Grimaldi

**Affiliations:** 1 Institute of Experimental Endocrinology and Oncology “G. Salvatore” (IEOS), National Research Council (CNR), 80131 Naples, Italy; 2 National Research Council (CNR), Piazzale Aldo Moro, 700185 Rome, Italy; 3 Steril Farma Srl, Via L. Da Vinci 128, 80055 Portici, Italy

**Keywords:** PARP10, Aurora-A, Mono-ADP-ribosylation, MARylation, PARPs, NAD, post-translational modification, G2/M transition, mitosis, cell cycle

## Abstract

**Simple Summary:**

Mono-ADP-ribosylating PARP enzymes are involved in the regulation of several cellular pathways, including cell cycle. The mono-ADP-ribosyltransferase PARP10 is frequently amplified in different tumor types, and has been shown to promote in vitro cellular proliferation and in vivo tumoral growth. The aim of our study was to investigate the role of PARP10 in the regulation of G2/M transition. We found that PARP10 is involved in the mono-ADP-ribosylation of Aurora-A, an important mitotic kinase, during the G2/M transition. Moreover, PARP10-catalyzed modification enhances the kinase activity of Aurora-A. Consistently, the depletion of PARP10 affects the timely recruitment of Aurora-A on centrosomes and mitotic spindle, causing a delay in mitotic entry. Our discovery provides novel details in the cellular functions exerted by PARP10.

**Abstract:**

Intracellular mono-ADP-ribosyltransferases (mono-ARTs) catalyze the covalent attachment of a single ADP-ribose molecule to protein substrates, thus regulating their functions. PARP10 is a soluble mono-ART involved in the modulation of intracellular signaling, metabolism and apoptosis. PARP10 also participates in the regulation of the G1- and S-phase of the cell cycle. However, the role of this enzyme in G2/M progression is not defined. In this study, we found that genetic ablation, protein depletion and pharmacological inhibition of PARP10 cause a delay in the G2/M transition of the cell cycle. Moreover, we found that the mitotic kinase Aurora-A, a previously identified PARP10 substrate, is actively mono-ADP-ribosylated (MARylated) during G2/M transition in a PARP10-dependent manner. Notably, we showed that PARP10-mediated MARylation of Aurora-A enhances the activity of the kinase in vitro. Consistent with an impairment in the endogenous activity of Aurora-A, cells lacking PARP10 show a decreased localization of the kinase on the centrosomes and mitotic spindle during G2/M progression. Taken together, our data provide the first evidence of a direct role played by PARP10 in the progression of G2 and mitosis, an event that is strictly correlated to the endogenous MARylation of Aurora-A, thus proposing a novel mechanism for the modulation of Aurora-A kinase activity.

## 1. Introduction

ADP-ribosylation is a reversible, multifunctional, post-translational modification consisting in the transfer of an ADP-ribose (ADPr) moiety from nicotinamide adenine dinucleotide (NAD+) to target substrates, with the release of nicotinamide (NAM) [1,2,3]. Originally described as a virulent mechanism of certain bacterial toxins that infect target cells, as shown for Cholera toxin (CTx) and Diphtheria toxin (DTx) [4,5], ADP-ribosylation has since been discovered to also be exerted by different ADP-ribosyltransferases (ARTs) in eukaryotes [3,6]. In humans, 17 intracellular diphtheria toxin-like ARTs (ARTDs) and 5 extracellular cholera toxin-like ARTs (ARTCs) account for the larger part of endogenous ADP-ribosylation [7]. Intracellular ARTDs are divided into mono-ARTs (PARP3, PARP4, PARP6–12 and PARP14–16) and poly-ARTs (PARP1, PARP2, Tankyrase-1 and -2), according to their ability to transfer a single ADPr (MARylation) or multiple ADPr units (PARylation) onto acceptor proteins, respectively [8].

Collectively, the PARP enzymes regulate a wide variety of cellular processes such as DNA damage response and repair, telomere function, intracellular trafficking, cellular stress, viral response and cell cycle [9,10,11,12,13,14]. Regarding this last process, the first evidence of a regulatory role for PARylation in DNA synthesis was provided in 1970, followed by different studies showing that poly-ADP-ribose (PAR) synthesis takes place even in other phases of the cell cycle [15,16]. The mammalian cell cycle is a multi-step process initiated by mitogenic stimuli that lead to the activation, degradation or post-translational modifications of specific proteins, with the final aim of ensuring the doubling of cellular components and their segregation in two identical cells. Differently from PARylation, the discovery that MARylating enzymes (PARP3, PARP6, PARP8, PARP10 and PARP14) contribute to the regulation of the different phases of the cell cycle is relatively recent. Indeed, unlike the large body of studies focused on the PARylating enzyme PARP1, the number of reports pinpointing the cellular activities of mono-ARTs has been growing only over the last decade [17,18,19,20,21,22,23,24,25,26,27,28,29,30,31,32,33,34,35,36,37].

PARP10 has been the first described MARylating ARTD enzyme [36,38]. PARP10 shuttles between the nucleus and the cytoplasm, catalyzing the MARylation of different protein substrates [30,34,39,40,41,42,43]. Different studies have shown its involvement in inflammation, apoptosis, DNA repair, metabolism and cell cycle [39,41,44,45,46,47]. Besides the characteristic PARP catalytic domain, PARP10 possesses two distinctive ubiquitin interacting motifs (UIMs) that confer specificity to its cellular functions, such as the regulation of NF-κB signaling and recruitment into p62/SQSTM1-positive structures [30,34]. The functions exerted by PARP10 in the regulation of the cell cycle are at different levels. In particular, the phosphorylation of PARP10 during the late G1-S phase by G1 kinase CDK2 has been proposed as a regulatory mechanism to support cell growth, while the recruitment of PARP10 at the DNA-damage sites by PCNA has been shown to improve the DNA damage tolerance during the S-phase [39,41].

Typically, most of the mammalian signaling pathways, including those involved in the cell cycle, are regulated by integrating protein PTMs, such as phosphorylation, acetylation, ubiquitination, SUMOylation and ADP-ribosylation. Thanks to the latest advancements in the ADP-ribosylation field, emerging studies show that the inter-regulation between kinases and ARTs is a recurring mechanism in the control of many signaling networks, although the overall picture is still far from being fully defined [23,27,39,42,48]. Interestingly, PARP10 has been shown to MARylate some of the kinases involved in the regulation of the cell cycle both in vitro and in cells, such as the G1 kinases CDK2 and CDK7 or the G2/M kinases PLK1 and Aurora-A [42,44,49]. However, the impact of their MARylation in the context of cell cycle regulation remains unknown.

Here, we describe the role of PARP10 in the regulation of G2/M transition and shed light on the crosstalk with the mitotic kinase Aurora-A. In particular, we show that depletion of PARP10 causes defects in the progression of the G2/M phase of the cell cycle. We demonstrate that endogenous Aurora-A is MARylated in mammalian cells and that this modification increases during the G2/M transition of the cell cycle, in a PARP10-dependent manner. Furthermore, we show that PARP10-mediated MARylation enhances the Aurora-A catalytic activity, stimulating the kinase’s autophosphorylation on T288. Finally, we find that PARP10 is critical for the recruitment of Aurora-A on the centrosomes, thus guaranteeing an efficient progression through the G2/M phase of the cell cycle.

## 2. Materials and Methods

### 2.1. Molecular Cloning and Site-Directed Mutagenesis

Human PARP10, inserted in pCMV6-XLS (Clone SC103603, Origene, Rockville, MD, USA), was cloned into the EcoRI/BamHI sites of pcDNA3-N-3xHA vector by PCR, using primers: 5’-GATCCGGAATTCATGGTTGCAATGGCGGAGGCAGAG-3’ and 5’-GATCGCGGATCCTTAAGTGTCTGGGGAGCGGCCCC-3’ together with PfuUltra High Fidelity DNA Polymerase AD (Agilent, Santa Clara, CA, USA). Mutant pcDNA3/hPARP10G888W 3xHA was obtained by PCR and using primers: 5’-CAGGTGCTGTACCACTGGACGACGGCACCGGCAG-3’ and 5’-TGCCGGTGCCGTCGTC CAGTGGTACAGCACCTG-3’, together with PfuUltra High Fidelity DNA Polymerase AD (Agilent, Santa Clara, CA, USA). Both PARP10 and PARP10-G888W were transferred into the EcoRI/XhoI sites of pGEX4T1 (Cytiva, Marlborough, MA, USA) for expression in *E. coli* via PCR using primers 5’-GATCCGGAATTCATGGTTGCAATGGCGGAGGCAGAGGC-3’ and 5’-GATCCGCTCGAGTTAATGTTGGGGAGCGGCCCGG-3’ together with PfuUltra High Fidelity DNA Polymerase AD (Agilent, Santa Clara, CA, USA). The sequences of all the constructs were verified by sequence analysis.

### 2.2. Protein Purification

GST-PARP10 and GST-PARP10-G888W were expressed in BL21 (DE3) pLysS, by inducing with 0.2 mM IPTG for 20 h at 20 °C, and lysed in: 1× PBS; 5 mM DTT; 1 mg/mL Lysozyme; 1 mM PMSF; 1% Triton X-100; and 1× cOmplete EDTA-free Protease Inhibitor Cocktail (Roche, Basel, Switzerland) for 30 min at 4 °C. Following sonication, the cleared lysate was incubated for 2 h with 1 mL washed Glutathione Sepharose 4B (Cytiva, Marlborough, MA, USA) in 1× PBS, 1 mM DTT and 100 mM PMSF, before washing thoroughly with the same buffer and eluting the recombinant protein with: 50 mM Tris-HCl (pH 8.0); 20 mM reduced Glutathione (Merck, Darmstadt, Germany); 100 mM NaCl; and 100 mM PMSF. Peak fractions were dialyzed against: 50 mM Tris-HCl (pH 8.0); 100 mM NaCl; 1 mM DTT; 100 mM PMSF; and 10% Glycerol; before further purification on a 1 mL MonoQ column (Cytiva, Marlborough, MA, USA) using a linear gradient to 500 mM NaCl. The protein eluted at approximately 400 mM NaCl.

### 2.3. Cell Culture and Cell Cycle Synchronization

HeLa-M (RRID:CVCL_R965) and HepG2 cell lines (RRID:CVCL_0027) were originally obtained from the laboratory of Dr. Alberto Luini (IEOS, National Research Council, Naples, Italy) and were maintained in DMEM medium. T47D (RRID:CVCL_0553), A375 (RRID:CVCL_0132) and U2OS (RRID:CVCL_0042) cell lines were obtained from ATCC and were cultured in RPMI, DMEM-F12 and McCoy’s medium, respectively. All media were supplemented with 10% FBS (Biochrom), 100 U/mL penicillin/streptomycin and 2 mM L-glutamine (Thermo Fisher Scientific, MA, USA). All cells were maintained at 37 °C in a humidified atmosphere of 5% CO_2_. For cell cycle synchronization, the population was treated twice overnight with 2 mM thymidine (Merck, Darmstadt, Germany); after each incubation with thymidine, the cells were washed once in sterile PBS and grown in the complete medium for 10 h.

### 2.4. PARP10 KO HeLa Cell Line Generation

The genetic disruption of PARP10 in HeLa cells was performed via CRISPR-Cas9 gene editing. The single-guide RNAs (sgRNAs) targeting exon 2 of human PARP10 gene (forward: 5’-caccgAAAACCGCCGACGCTCTGGA-3’ and reverse: 5’-aaacTCCAGAGCGTCGGCGGTTTTc-3’) were designed using the CRISPick tool (https://portals.broadinstitute.org/gpp/public/analysis-tools/sgrna-design accessed on 21 September 2020). sgDNA oligonucleotides were annealed and cloned into the BbsI digested pSpCas9 (BB)-2A-GFP vector; SpCas9 (BB)-2A-GFP (PX458) was a gift from Feng Zhang (Addgene plasmid #48138). HeLa cells were transfected with 5 μg of pSpCas9 (BB)-2A-GFP plasmid containing the respective sgRNA using Lipofectamine LTX (Life Technologies, Carlsbad, CA, USA). Following 24 h incubation, the GFP-positive cells were individually isolated by fluorescence-activated cell sorting (FACS). The clones were expanded and validated by Western blotting and the Sanger sequencing of PCR amplicons was generated using specific primers (GP10F: 5’-GGGAGCATTGAGGACACACCTTGGAGG-3’; and GP10R: 5’-GCGGGGCAGGATGTCAGGCATTAG-3’). The generation of PARP10 KO-rescue HeLa cell lines was obtained by the transient transfection of pcDNA3/hPARP10 3xHA (wild-type) or pcDNA3/hPARP10G888W 3xHA (mutant) for 24 h and following selection with Neomycin. The selected cells were individually isolated, and the resulting clones were validated for the re-expression of PARP10 by Western blotting.

### 2.5. Antibodies and Reagents

For Western blot analysis, the following antibodies were used: rat anti-PARP10 (sc-53858) from Santa Cruz Biotechnology (Santa Cruz, CA, USA); mouse anti-Aurora-A (15815719) from Thermo Fisher Scientific (Waltham, MA, USA); rabbit anti-phospho-T288 Aurora-A (#3079) and rabbit anti-MAR/PAR (#83372) from Cell Signalling Technology (Beverly, MA, USA); and mouse anti-GAPDH (ab8245) from AbCam (Cambridge, UK). Secondary antibodies were obtained from Merck (Darmstadt, Germany). For immunofluorescence analysis, the following antibodies were used: rabbit anti-Aurora-A (#14475) from Cell Signalling Technology (Beverly, MA, USA); mouse anti-Pericentrin (ab28144) from AbCam (Cambridge, UK); and rabbit anti-phospho-Ser10 Histone-H3 (06/570) form Merck (Darmstadt, Germany). Hoechst nuclear dye and RNAse were purchased from Thermo Fisher Scientific (Waltham, MA, USA). Propidium Iodide (PI), Thymidine, PJ34, β-NAD^+^ and ATP were sourced from Merck (Darmstadt, Germany). OUL35 [47] was kindly provided by Prof. Lari Lehtio (University of Oulu, Finland) and Prof. Oriana Tabarrini (University of Perugia, Italy).

### 2.6. Flow Cytometry

Trypsinized HeLa cells were pelleted, washed in ice-cold PBS, resuspended in ice-cold ethanol (while vortexing) and then incubated overnight at 4 °C. On the following day, samples were centrifuged at 500× *g* for 5 min, the ethanol was removed, and the cells were washed in ice-cold PBS and incubated with 50 μg/mL propidium iodide for 30 min in the presence of RNAse. The cells were then analyzed using the Becton Dickinson (BD) FACSCantoA instrument.

### 2.7. Immunofluorescence and Confocal Microscopy

The cells were fixed in 4% paraformaldehyde for 10 min at room temperature (RT), washed three times in PBS and incubated for 30 min at RT in blocking solution (0.5% bovine serum albumin; 50 nM NH4Cl in PBS; pH 7.4; 0.1% saponin and 0.02% sodium azide). The cells were subsequently incubated at 4 °C with the indicated antibodies diluted in blocking solution. Following an overnight incubation, the cells were washed three times in PBS and incubated with a fluorescent-probe-conjugated secondary antibody for 30 min at RT. Alexa Fluor 488- or 568-conjugated anti-rabbit or anti-mouse donkey antibodies were used at a dilution of 1:400 in blocking solution. Following immunostaining, the cells were washed three times in PBS and twice in sterile water. The coverslips were then mounted on glass-microscope slides with Mowiol. The images were taken using a Zeiss-LSM 700 confocal microscope. The optical confocal sections were taken at 1 Airy Unit. The image analysis was performed using the open source image processing software ImageJ2 (version 2.9.0, National Institute of Health, Bethesda, Maryland).

### 2.8. Macro Domain-Based Pull-Down Assay 

The procedure to obtain the Af1521 *macro* domain resin has been described previously [50]. For pulldown assays, HeLa cells were washed three times in ice-cold PBS and solubilized in RIPA buffer (50 mM Tris HCl; pH 7.5; 150 mM NaCl; 1% NP-40; 0.5% deoxycholate and 0.1% SDS; supplemented with protease inhibitors), under constant rotation for 30 min at 4 °C. The mixtures were clarified by centrifugation at 15,000× *g* for 10 min at 4 °C, the supernatants were recovered, and the protein concentration was evaluated using the Bradford Protein Assay (Bio-Rad). The total lysates (0.5–1 mg) were then incubated with 20 µL of a 10 µg/µL GST cross-linked wild-type macro domain resin at 4 °C. Following overnight incubation, beads were centrifuged at 500× *g* for 5 min to recover the proteins bound to the Af1521 *macro* domain. The resins were then washed 3 times with RIPA buffer and another 2 times in the same buffer without detergents. At the end of the washing steps, the resins were resuspended in 50 µL SDS sample buffer, boiled, analyzed by 10% SDS/PAGE and transferred onto nitrocellulose for Western blotting.

### 2.9. siRNA-Mediated Knockdown

The siRNA oligos targeting hPARP10 (Dharmacon, L-014997-00-0010) or non-targeting siRNAs (Dharmacon, D-001810-01-05), were transfected in HeLa and T47D cells at a final concentration of 20 or 50 nM using Lipofectamine RNAiMAX reagent (Invitrogen, 13778150), according to the manufacturer’s instructions.

### 2.10. In Vitro ADP-Ribosylation-Kinase Assay

The purified, GST-tagged, full-length PARP10 wild-type or the catalytically inactive mutant (G888W) (80 ng) was incubated with 80 ng purified recombinant wild-type histidine-tagged Aurora-A (Enzo Life Sciences) in ADP-ribosylation Buffer (50 mM Tris-HCl pH 7.4; 2 mM DTT; 500 μM MgCl_2_; 100 μM NA^+^; and 1 × cOmplete EDTA-free Protease Inhibitor Cocktail), at 37 °C for 120 min. At the end of the reaction, 1 μM OUL35 was added to the mixture for 10 min. Next, the kinase reaction was performed adding 2× kinase buffer (50 mM Tris pH 7.4, 3 mM MgCl_2_, 2 mM DTT and 1 mM ATP, in the presence of 1 × cOmplete EDTA-free Protease Inhibitor Cocktail and 1× PhosStop phosphatase inhibitors) to the mixture, for 60 min at 37 °C. At the end of the incubation, the reaction was stopped by adding a 2 × SDS-sample buffer. The samples were boiled at 95 °C for 5 min and resolved by SDS-PAGE, blotted onto nitrocellulose and analyzed by Western blotting using the indicated antibodies.

### 2.11. Western Blot Analysis

The nitrocellulose membranes were blocked for 1 h at RT in Tris-Buffered Saline (TBS; Bio-Rad Laboratories, Hercules, CA, USA) with 0.05% Tween-20 (TBS-T; Merck, Darmstad, Germany) containing 5% non-fat dry milk (Merck, Darmstad, Germany). The primary antibodies were diluted in blocking solution and incubated with membranes overnight at 4 °C. After washing with TBS-T, the membranes were incubated with an appropriate HRP-conjugated secondary antibody diluted in 5% non-fat, dry milk in TBS-T for 45 min at RT. The membranes were washed extensively with TBS-T before chemiluminescent detection using the ECL Western Blotting Detection Reagents (Merck, Darmstad, Germany; Cytiva, Marlborough, MA, USA) and X-ray film (Fujifilm, Tokyo, Japan) or ChemiDoc MP (Bio-Rad Laboratories, Hercules, CA, USA). Densitometry analysis was performed using ImageJ2 software.

### 2.12. Statistical Analysis

*p*-values were calculated comparing control and each treated group individually, using Student’s t test. All statistical parameters are listed in the corresponding figure legends.

## 3. Results

### 3.1. PARP10 Regulates G2/M Transition

**Figure 1:**
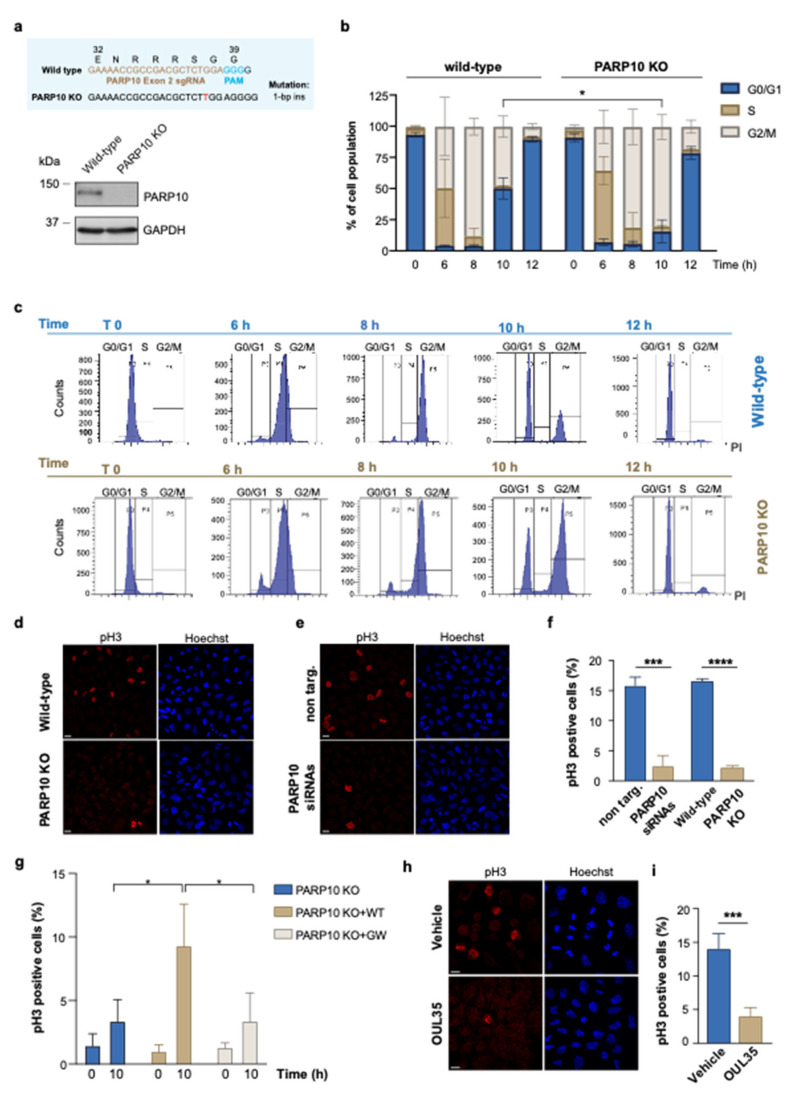
Depletion of PARP10 impairs the G2/M transition. (**a**) PARP10 KO HeLa cell line was generated by CRISPR/Cas9 approach. The resulting KO clone was validated by Sanger sequencing (upper panel) and Western blot analysis (lower panel). (**b**,**c**) Wild-type and PARP10 KO HeLa cells were synchronized by using double thymidine blocks, harvested at the indicated time points, and analyzed by flow cytometry using propidium iodide staining. Quantification data are means (±S.D.) from three independent experiments. (**d**,**e**) PARP10 KO HeLa cells or HeLa cells transfected for 48 h with PARP10 siRNA pool (PARP10 siRNAs) and their respective controls were synchronized as in (**b**), fixed 10 h hours after thymidine release and processed for immunofluorescence staining using pH3 antibody. Hoechst was used to label nuclei. Representative images are shown. (**f**) Quantification of the percentage of cells positive for pH3 staining. Data are means (±S.D.) from three independent experiments. (**g**) PARP10 KO HeLa cells and PARP10 KO cell lines stably expressing either the wild-type PARP10 or its catalytically inactive mutant (PARP10 G888W) were synchronized as in (**b**) and fixed 10 h after thymidine release. Cells were processed for immunofluorescence using pH3 antibody and percentage of cells positive for pH3 staining was quantified. Data are means (±S.D.) from three independent experiments. (**h**) Representative confocal microscopy images of synchronized HeLa cells treated for 6 h with 10 µM OUL35 or with vehicle alone (DMSO) and then fixed 10 h after thymidine release. Cells were processed for immunofluorescence staining using pH3 antibody. (**i**) Quantifications of the percentage of cells positive for pH3 staining. Data are means (±S.D.) from three independent experiments. Two-tailed Student’s *t*-tests were applied to the data (* *p* < 0.05; *** *p* < 0.001; **** *p* < 0.0001). Scale bars, 10 μm. Full pictures of the Western blots are presented in Figure S6.

Due to the manifold evidence pointing to the involvement of PARP10 during the progression of the cell cycle, we were prompted to investigate its complicity in the regulation of the G2/M transition [39,41,44,51]. We initially analyzed G2/M-enriched HeLa cells using a double thymidine block cell synchronization protocol (see Methods). As an experimental model, we generated HeLa cells depleted of PARP10 (PARP10 KO) with the use of the CRISPR/Cas9 technology (Figure 1a) [52].

Wild-type and PARP10 KO cells were harvested at various time points after cell synchronization and stained with propidium iodide to analyze cell-cycle progression by flow cytometry. As shown in Figure 1b,c, the PARP10 KO cells exhibited a delayed progression along the G2/M phase compared to wild-type cells, with a significant difference at 10 h (T10) after thymidine release. Following this delay, PARP10 KO cells can progress along the cell-cycle, although less efficiently, thus compensating for the absence of PARP10.

To further dissect the role of PARP10 in the G2/M phase, we analyzed the extent of mitosis by indirect immunofluorescence in control cells (wild-type and non targ.) and in PARP10 KO or siRNA-depleted HeLa cells probed with an antibody recognizing Histone H3 phosphorylated on serine 10 (pH3), which is a well-established marker of mitosis (Figure 1d,e and Figure S1a) [53]. Applying the synchronization protocol described above, we found a dramatic decrease in mitotic cells at T10 upon both the depletion and ablation of PARP10 compared to the controls (non-targ. and wild-type cells; Figure 1f). Similar results were obtained in a different cell model (Figures S1a and S2): the breast cancer cell line T47D was transiently devoid of PARP10 expression, further confirming the involvement of this MARylating enzyme during mitotic progression.

Additionally, with the aim to elucidate the role of PARP10 enzymatic activity in this process, we generated PARP10 KO cell lines stably expressing either the wild-type PARP10 or its catalytically inactive mutant (PARP10 G888W, see Figure S1b and Methods), and performed cell-cycle analysis by measuring the percentage of mitotic cells, over the total population, as described above. Our data show that the over-expression of the wild-type PARP10 in the PARP10 KO cells was able to rescue the mitotic entry defect, while the catalytically inactive PARP10 (PARP10 G888W) was not (Figure 1g). In parallel, the relevance of PARP10 catalytic activity in regulating cell-cycle progression was evaluated by analyzing the extent of mitosis in HeLa cells as well as in additional cancer cell lines, all expressing comparable levels of PARP10 protein (Figure S3a), upon PARP10 inhibitor treatment. To this end, we quantified the percentage of the mitotic population in HeLa (ovarian cancer), A375 (melanoma), U2OS (osteosarcoma), T47D (breast cancer) and HepG2 (hepatocellular carcinoma) cells treated with the PARP10 specific inhibitor OUL35 [47]. Strikingly, the data obtained showed that, in all cell lines tested, the pharmacological inhibition of PARP10 caused an impairment in the enrichment of mitotic cells compared to the vehicle-treated sample (Figure 1h,i and Figure S3b), a phenotype resembling PARP10-depletion.

Altogether, these results demonstrate that the MARylating activity of PARP10 is necessary for the proper execution of the G2/M phase during the cell cycle and appears to be a conserved mechanism of cell-cycle regulation, specifically acting at the G2/M boundary.

### 3.2. Aurora-A Is MARylated in HeLa Cells

The phenotype observed upon PARP10 depletion or inhibition led us to look for previously identified PARP10 targets that belong to the network regulating the G2/M transition of the cell cycle. As previously reported, the mitotic kinase Aurora-A is an in vitro substrate of PARP10 [44] and an ideal candidate for our next analyses. Evidence for the ADP-ribosylation of Aurora-A in cells has been formerly provided by different proteomic studies [54,55,56,57], all performed upon treatment with hydrogen peroxide, a well-known cell stressor and inducer of ADP-ribosylation. Although this approach allows for the identification of a wide number of ADP-ribosylation targets, it prevents them from being framed in the proper physiological context.

**Figure 2:**
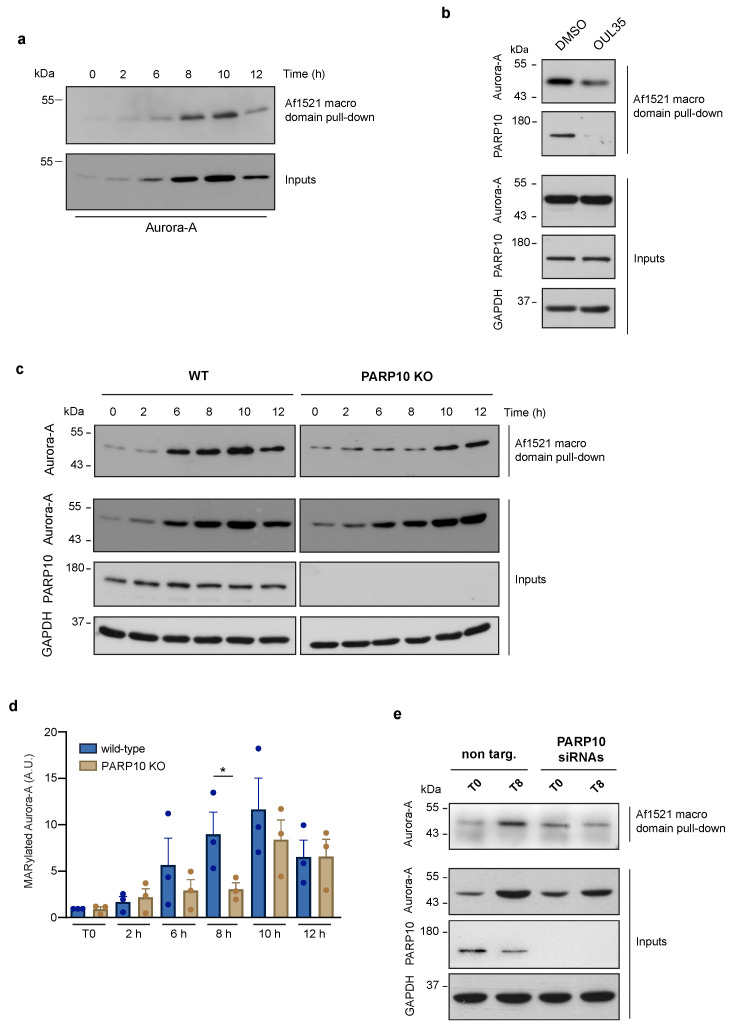
Aurora-A MARylation is dependent on PARP10 during the G2/M transition. MARylation levels of Aurora A were analyzed using the Af1521 macro domain-based pull-down assay on total cell lysates obtained from: (**a**) HeLa cells harvested at different time points (as indicated) after double thymidine block; (**b**) synchronized HeLa cells harvested at 8 h after release untreated (control) or treated with the PARP10 inhibitor OUL35 (10 µM, 2 h); (**c**) wild-type and PARP10 KO HeLa cells harvested at different time points (as indicated) after double thymidine block; (**d**) Quantification of MARylated Aurora-A from c. Data are means (±S.D.) from three independent experiments. Two-tailed Student’s t-tests were applied to the data (* *p* < 0.05). (**e**) Total lysates from synchronized HeLa cells treated (48 h) with non-targeting siRNA (non-targ.) or a pool of PARP10 siRNAs (PARP10 siRNAs), were harvested at 8 h after release and analyzed for Aurora-A ADP-ribosylation by Af1521 macro domain-based pull-down assay. Full pictures of the Western blots are presented in Figure S6.

To overcome this limitation, we chose to investigate the function of endogenous Aurora-A ADP-ribosylation in the regulation of the cell cycle, thus under physiologically non-stressing conditions. As for the other cell cycle proteins [58], levels of Aurora-A are dynamically regulated, increasing at the onset of the G2 phase, reaching a peak at the G2/M transition and decreasing dramatically when the next G1 phase is initiated [59]. Thus, by exploiting the Af1521 macro domain-based pull-down, a well-recognized approach for isolating ADP-ribosylated proteins, we probed the ADP-ribosylation of endogenous Aurora-A specifically during the G2/M transition. The HeLa cells previously arrested in the S-phase were thymidine-released and analyzed for both Aurora-A protein and MARylation levels at different time points after their release, as indicated (Figure 2a). As shown in Figure 2a (bottom), the Aurora-A protein level gradually augmented after release from the S-phase block, reaching its peak 8–10 h later. Interestingly, a comparable increase was observed for the ADP-ribosylated pool of Aurora-A, suggesting that newly synthesized Aurora-A is actively modified by one or more ARTs during cell-cycle progression (Figure 2a, upper blot). As expected, Aurora-A ADP-ribosylation was reduced when cells were treated with the broad PARP inhibitor PJ34 (Figure S4) [60], confirming the specificity of the binding of Aurora-A to the Af1521 macro domain.

### 3.3. Aurora-A MARylation Requires PARP10

The data showing the enhancement of Aurora-A ADP-ribosylation during the G2 phase prompted us to identify the endogenous ART enzymes involved in its modification. Based on our evidence and others’, we selected PARP10 as the leading candidate for the catalysis of the endogenous MARylation of Aurora-A. To validate our hypothesis, we analyzed ADP-ribosylation of Aurora-A in HeLa cells that were subjected to treatment with OUL35. As shown in Figure 2b, pharmacological inhibition of PARP10 caused a reduction in the MARylation of Aurora-A, evaluated by Af1521 macro domain pull-down, suggesting a direct and selective modification of Aurora-A by PARP10. To further investigate the functional relationship between these two proteins, we analyzed both total and ADP-ribosylated Aurora-A from the wild-type and PARP10 KO cells subjected to the synchronization protocol as above. Unlike parental cells, PARP10 KO cells showed a general decrease of MARylated Aurora-A, with a significant reduction at 8 h after release (Figure 2c,d), although a slight decrease in the total levels of Aurora-A (Figure 2c and Figure S5) can be observed at 8/10 h time points. To corroborate these results, HeLa cells were transiently depleted of PARP10 expression through siRNA-mediated silencing. As observed in PARP10 KO cells, PARP10 gene silencing caused a strong decrease in Aurora-A MARylation compared to the control cells 8 h after the G1/S block release (Figure 2e).

Collectively, our data demonstrate that Aurora-A is MARylated at steady-state in the absence of chemical stressors, and that its modification, as well as its protein level, increases during cell-cycle progression, with a peak specifically occurring at the G2/M boundary. Further, our data demonstrate that PARP10 drives the endogenous MARylation of Aurora-A during G2/M progression, thus underlining the importance of this PTM during this phase of the cell cycle.

### 3.4. PARP10-Mediated MARylation Enhances Aurora-A Catalytic Activity

**Figure 3:**
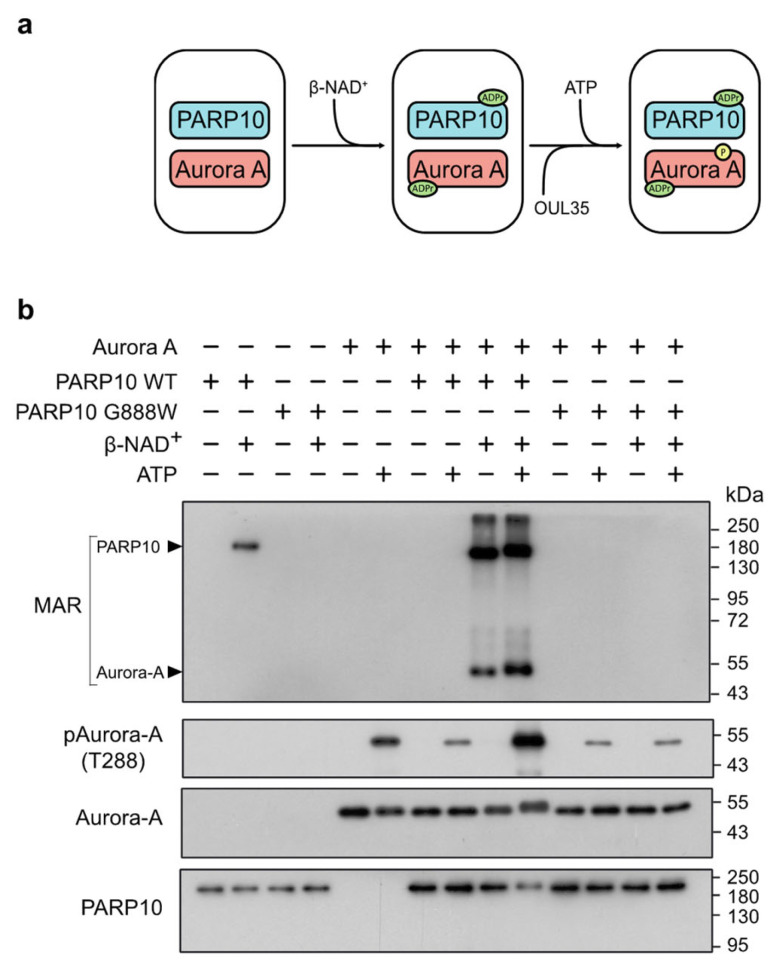
PARP10 enhances Aurora-A kinase activity in vitro. (**a**) Schematic illustration of the two-step ADP-ribosylation/kinase assay using purified GST-PARP10 or its catalytically inactive mutant (GST-PARP10-G888W) and His-Aurora-A. (**b**) Two-step ADP-ribosylation/kinase assay. ADP-ribosylated proteins were detected using an anti-MAR/PAR antibody. Phosphorylated Aurora-A was evaluated using an anti-phospho-T288 Aurora-A antibody. Levels of PARP10 and Aurora-A were controlled by using an anti-PARP10 antibody and an anti-Aurora-A antibody, respectively. Results shown are representative of three independent experiments. Full pictures of the Western blots are presented in Figure S6.

Intrigued by the consequences of PARP10 ablation on the endogenous ADP-ribosylation of Aurora-A and on G2/M transition, we next investigated whether PARP10-mediated MARylation could influence the kinase activity of Aurora-A by setting up a two-step in vitro assay, consisting of an ADP-ribosylation assay followed by a kinase assay (see Methods), performed by incubating the GST-tagged wild-type or dead mutant (G888W) PARP10 with His-tagged Aurora-A (Figure 3a).

Specifically, protein ADP-ribosylation mediated by PARP10 was evaluated using a MAR/PAR specific antibody, while the catalytic activity of Aurora-A was followed using an antibody recognizing the phosphorylated threonine 288 (T288) residue, the primary site for Aurora-A auto-phosphorylation [61]. As expected, the wild-type PARP10 was able to MARylate both Aurora-A and itself, whereas PARP10 G888W was not (Figure 3b, MAR). Strikingly, the auto-phosphorylation of Aurora-A on T288 was largely enhanced by PARP10-catalyzed MARylation compared to non-MARylated samples (Figure 3b, pAurora-A), demonstrating that the MARylation catalyzed by PARP10 enhances the kinase activity of Aurora-A in vitro.

### 3.5. PARP10 Depletion Affects Aurora-A Recruitment on Centrosomes and Their Maturation

Aurora-A regulates mitotic entry and progression, with functions linked to both the maturation of centrosome and the genesis of the mitotic spindle, events that rely on the phosphorylation of different target proteins initiated by the Aurora-A auto-phosphorylation on T288. In particular, the recruitment of activated Aurora-A on the centrosomes is a key event in the regulation of centrosomal substrates and mitosis execution [62].

**Figure 4:**
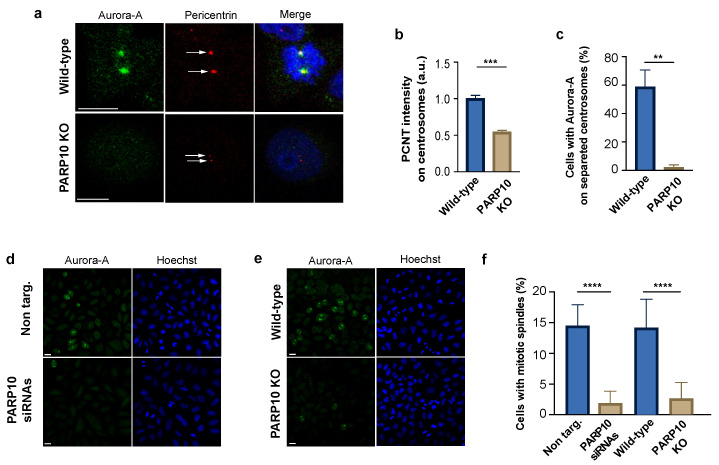
PARP10 favors Aurora-A centrosomal recruitment. (**a**) Representative images of synchronized wild-type and PARP10 KO HeLa cells fixed 10 h after thymidine release and processed for immunofluorescence using Aurora-A (green) and pericentrin (red) antibodies. Arrows indicate duplicated centrosomes. (**b**) Measurements of the fluorescence intensity of pericentrin recruited on centrosomes (A.U.). (**c**) Quantification of the percentage of cells positive for Aurora-A staining on separated centrosomes. (**d**,**e**) Representative microscopy confocal images of HeLa cells KO for PARP10 or treated with PARP10 siRNAs (48 h) and their respective controls processed for immunofluorescence using Aurora-A (green). (**f**) Quantification of the percentage of cells showing Aurora-A positive mitotic spindles. Data are means (±S.D.) from three independent experiments. Nuclei were labelled with Hoechst staining. Two-tailed Student’s *t*-tests were applied to the data (** *p* < 0.01; *** *p* < 0.001; **** *p* < 0.0001). Scale bars, 10 μm.

Using indirect immunofluorescence, we analyzed the centrosome maturation checking the localization of Aurora-A and the amount of centrosomal protein pericentrin (PCNT), a protein that participates in the establishment of a centrosomal matrix that is important for the recruitment and activation of mitotic kinases. The synchronized wild-type and PARP10 KO cells were fixed 10 h after the thymidine washout, labelled with corresponding antibodies and examined by confocal microscopy. Importantly, in PARP10 KO cells, we observed a strongly reduced staining of PCNT on centrosomal structures (Figure 4a,b) and a decrement in the percentage of centrosomes that were positive for Aurora-A (Figure 4a,c), likely due to the observed delay in Aurora-A MARylation (Figure 2c,d) and the following recruitment on centrosomes. Nevertheless, the duplication and separation of the centrosomes were evident, although less pronounced in cells lacking PARP10. This behavior excludes a detrimental effect of the PARP10 depletion on G2 entry (Figure 4a, arrows) [63].

Finally, we examined the localization of Aurora-A on the mitotic spindle, a cellular structure established after the centrosome segregation to the cell poles deputed to the separation of chromosomes into two daughter cells during mitosis. In this case both siRNA-depleted and PARP10 KO cells were synchronized, fixed at 10 h after release and then analyzed by confocal microscopy (Figure 4d,e). We found that about 15% of cells in the control samples (non-targ. and wild-type) displayed a mitotic spindle labelled with Aurora-A. Conversely, the samples lacking or depleted of PARP10 bared a dramatic reduction in the fraction of cells showing mitotic spindles (Figure 4f), likely as a consequence of altered PARP10-mediated Aurora-A activation.

Taken together, our results show that MARylation of Aurora-A catalyzed by PARP10 enhances the autophosphorylation of the kinase on T288 and that, possibly, this is preparatory for the activation of Aurora-A and its following recruitment on the centrosomes, an event which is critical for the establishment of the mitotic spindle and execution of the whole mitotic process.

## 4. Discussion

In this study, we demonstrate that the mono-ADP-ribosyltransferase PARP10 contributes to the transition from the G2-phase to mitosis, and that MARylation of the mitotic kinase Aurora-A by PARP10 is required to stimulate the activity of the kinase and its recruitment to centrosomes, thus describing a novel role for PARP10 in the progression of the cell cycle. Further, we propose this PTM as a novel layer in the regulation of Aurora-A, required for a proper activation of the kinase, with important implications in centrosome function and mitosis progression.

The mammalian cell cycle is divided into interphase—which is composed of G1, S and G2-phase—and mitosis. The duplication of genomic DNA and the segregation of chromosomes into two daughter cells are confined into S-phase and mitosis, whereas during the gap-phases G1 and G2, the cell increases its mass, coordinates growth signals and makes itself available for cell division [64].

Different studies have pointed to the involvement of endogenous ADP-ribosylation reactions in the regulation of cell cycle progression [11,14]. Both members of poly-ARTs (PARP1, PARP2 and TNKS1) and mono-ARTs (PARP3, PARP6, PARP7, PARP10 and PARP14) regulate the different phases of the cell cycle, ranging from G1 to mitosis, through the transcriptional or post-translational regulation of different protein substrates [11,31,32,35,39,41,51,65,66,67,68,69,70,71,72,73]. Our data show a novel task for PARP10 in the progression of the cell cycle in addition to those exerted in G1 and S-phase, and point to a novel layer in the regulation of Aurora-A, a critical player in centrosome function and mitosis.

The mitotic kinase Aurora-A regulates the maturation of the centrosomes and the formation of the mitotic spindle [59,74,75,76]. The mechanisms involved in the spatio-temporal regulation of Aurora-A rely on the binding of co-factors that allosterically regulate Aurora-A, either inducing (CEP192, TPX2 and Ajuba), or not (Bora), its auto-phosphorylation on threonine 288 (Thr288), a residue positioned within the activation loop that is generally associated with the enhancement of its kinase activity [77,78,79,80,81]. Nevertheless, in some cases the phosphorylation on Thr288 is necessary but not sufficient to fully activate Aurora-A [82]. Indeed, phosphorylation, acetylation and SUMOylation on alternative residues by other enzymes are important to regulate Aurora-A activity and functions [61,83,84,85,86,87,88]. The biochemical analysis of PARP10-catalyzed MARylation on Aurora-A enzymatic activity shows that this PTM favors the activation of the kinase, as demonstrated by the inefficacy of the PARP10 catalytically inactive mutant (Figure 3b). Intriguingly, previous data reporting on the phosphorylation of PARP10 by other cell cycle kinases (CDK2, PLK1) point to the possibility of a feedback mechanism initiated by the MARylation of Aurora-A that, once activated, could in turn phosphorylate PARP10, thus affecting its transferase activity [39,42]. Collectively, our findings introduce MARylation as a novel accessory PTM capable of stimulating the catalytic activity of Aurora-A.

Functionally, we show that impairment of PARP10 activity by genetic manipulation or drug inhibition causes a delay in the recruitment of the Aurora-A on the centrosomes and the subsequent entry into mitosis (Figure 1), without affecting centrosomal separation (Figure 4a). Since a similar defect has been observed upon the inhibition of Aurora-A by the injection of a specific antibody during late G2 [88,89], it could be envisaged that the PARP10-catalyzed MARylation of Aurora-A is limited to this short time window, providing an interpretation to the delay, and not arrest, in the cell cycle progression observed in our study. In this context, it cannot be excluded that activity of other MARylating enzymes (e.g., PARP3) partially contribute to the modification and activation of Aurora-A [66], as inferred from cells depleted of PARP10, thus overcoming this defect, otherwise lethal, and guaranteeing the progression of the cell cycle.

It is worth mentioning that the PARP10 MARylation of Aurora-A in cells has been previously shown to have an inhibitory effect on the function of Aurora-A. While this may appear to be in contrast with our findings, it should be noted that Aurora-A was analyzed in the context of the regulation of tumor growth and invasion in non-synchronized cells [49]. Consequently, from these two sets of data, it could be concluded that PARP10 acts differently on Aurora-A depending on the specific cell process (e.g., tumor spreading vs. cell cycle regulation), leading to the specific regulation of different functions. While the ADP-ribosylation of Aurora-A has so far been reported as a consequence of oxidative stress and upon protein over expression [49,54,55,56,57], this report demonstrates that the endogenous ADP-ribosylation of Aurora-A can occur under physiological conditions and, most importantly, couple this PTM to the G2/M phase transition during the cell cycle (Figure 2a).

This study, along with others conducted by our group [23,90,91], extends our knowledge on non-stress related functions regulated by ARTs, and on the physiology of this PTM.

## 5. Conclusions

The pathway analyzed in this study acquires an additional value when considering that both PARP10 and Aurora-A are known oncogenes, and their overexpression causes dysregulation in cell growth and cell division, respectively. In particular, the multiple tumoral types showing overexpression of Aurora-A are characterized by mitotic defects such as centrosome abnormalities and chromosome instability (CIN), which favor cancer progression. In this respect, growing interest has been focused on the development of anti-cancer drugs targeting Aurora-A [92,93,94]. Nevertheless, treatment with Aurora-A inhibitors in the clinic show low tolerability due to the function of this kinase in highly proliferating cells, such as bone marrow cells and epithelial cells [95]. Moreover, the onset of drug-resistance due to prolonged treatment with these molecules could represent an important limitation to their therapeutic efficacy [96].

Considering the novel role held by PARP10 in Aurora-A activation during the cell cycle, we envisage the use of PARP10 inhibitors, solely or in combination with Aurora-A inhibitors, as a potential therapy for the treatment of Aurora-A overexpressing tumors.

## Data Availability

The data presented in this study are available in this article (and Supplementary Materials).

## Supplementary Materials

The following supporting information can be downloaded at: https://www.mdpi.com/article/10.3390/cancers14215210/s1, Figure S1: (a) T47D and HeLa cells were transiently transfected with non-targeting (non-targ.) or a pool of PARP10 siRNAs for 48 h. PARP10 depletion levels were detected using a PARP10 antibody on total cell lysates. Detection of actin was used as loading control. (b) PARP10 protein levels detected in the different cell lines, as indicated. Detection of GAPDH was used as loading control. WT PARP10 clone #6 and G888W PARP10 clone #5 were selected for comparable PARP10 protein levels and used for all following rescue experiments; Figure S2: Synchronized T47D cells were transfected with non-targeting (non-targ.) or a pool of PARP10 siRNAs for 48 h and synchronized by using double thymidine blocks; Figure S3: (a) PARP10 protein levels detected in total lysates from different cell lines, as indicated. Detection of actin used as loading control. (b) A375, U2OS, T47D and HepG2 cells were synchronized by using double thymidine blocks and treated with vehicle alone (DMSO) or with 10 μM OUL35 during the second thymidine release; Figure S4: MARylation levels of Aurora A were analyzed using the Af1521 macro domain–based pull-down assay on total cell lysates obtained from synchronized HeLa cells harvested at 8 h after thymidine release, left untreated (control) or treated with the PARP inhibitor PJ34 (50 μM); Figure S5: Quantification of Aurora-A protein levels during cell cycle progression, in wild-type and PARP10 KO HeLa cells; Figure S6: Original blots from all performed Western blots.
