# Supplementary material for: PARP10 Mediates Mono-ADP-Ribosylation of Aurora-A Regulating G2/M Transition of the Cell Cycle
Source: Cancers (Basel). 2022 Oct 24;14(21):5210. doi: 10.3390/cancers14215210 (PMC9659153; doi:10.3390/cancers14215210)
Supplement: Supplementary file 1 [file cancers-14-05210-s001.zip › cancers-1979306-supplementary.pdf]

a)

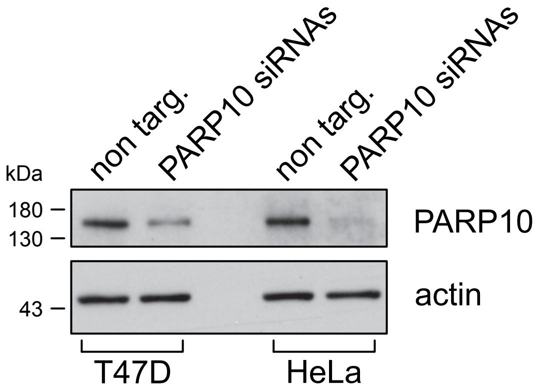

b)

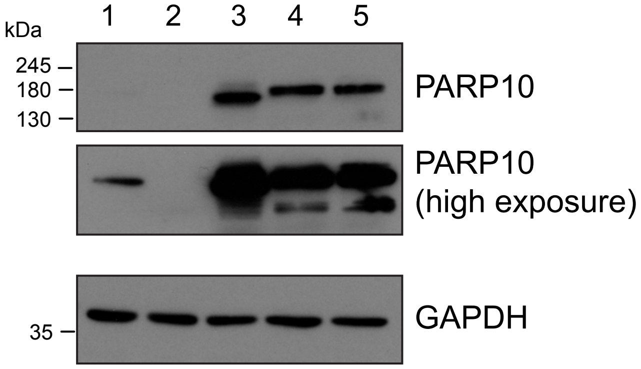

1: parental HeLa
2: PARP10 KO
3: PARP10 KO + WT PARP10 clone #3
4: PARP10 KO + WT PARP10 clone #6
5: PARP10 KO + G888W PARP10 clone #5

Figure S1
a) T47D and HeLa cells were transiently transfected with non targeting (non targ.) or a pool of PARP10 siRNAs for 48 h. PARP10 depletion levels were detected using a PARP10 antibody on total cell lysates. Detection of actin was used as loading control.
b) PARP10 protein levels detected in the different cell lines, as indicated. Detection of GAPDH was used as loading control. WT PARP10 clone #6 and G888W PARP10 clone #5 were selected for comparable PARP10 protein levels and used for all following rescue experiments.

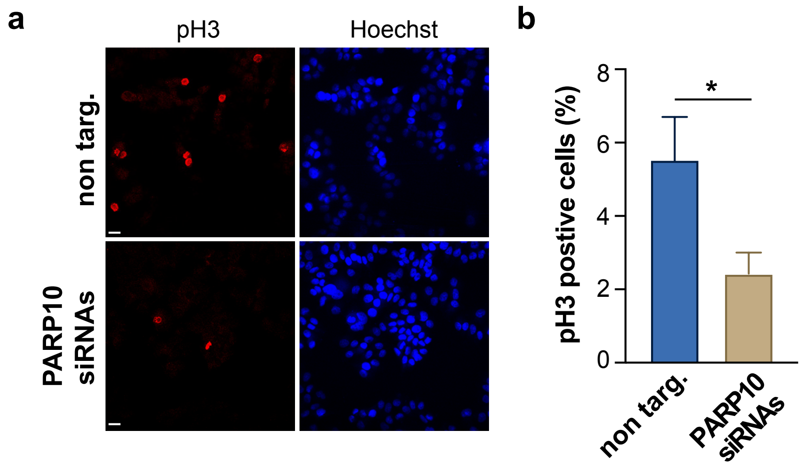

Figure S2
Synchronized T47D cells were transfected with non targeting (non targ.) or a pool of PARP10 siRNAs for 48 h and synchronized by using double thymidine blocks. After 10 hours after the second thymidine release, cells were fixed and processed for immunofluorescence staining using pH3 antibody. Hoechst was used to label nuclei. (a) Representative images are shown. (b) Quantification of the percentage of cells positive for pH3 staining. Data are means (± S.D.) from three independent experiments.

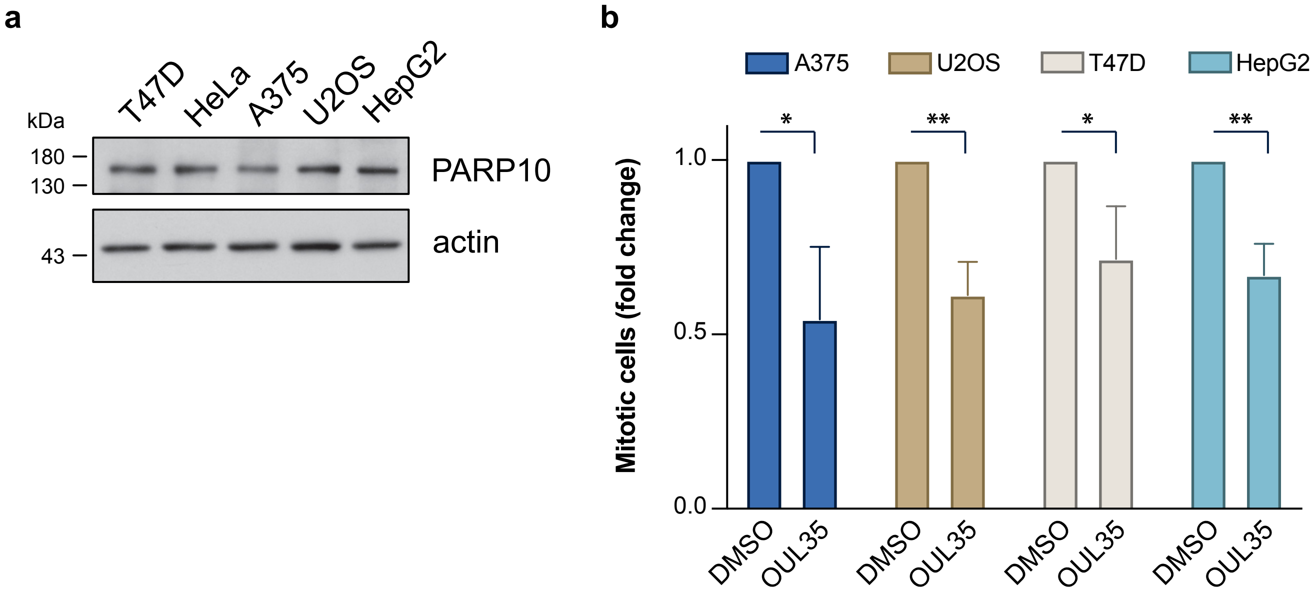

Figure S3
(a) PARP10 protein levels detected in total lysates from different cell lines, as indicated. Detection of actin used as loading control.
(b) A375, U2OS, T47D and HepG2 cells were synchronized by using double thymidine blocks and treated with vehicle alone (DMSO) or with 10 μM OUL35 during the second thymidine release. After 10 hours, cells were fixed and processed for immunofluorescence staining using pH3 antibody; Hoechst was used to label nuclei. The graph shows quantifications of the percentage of cells positive for pH3 staining. Data are means (± S.D.) from three independent experiments.

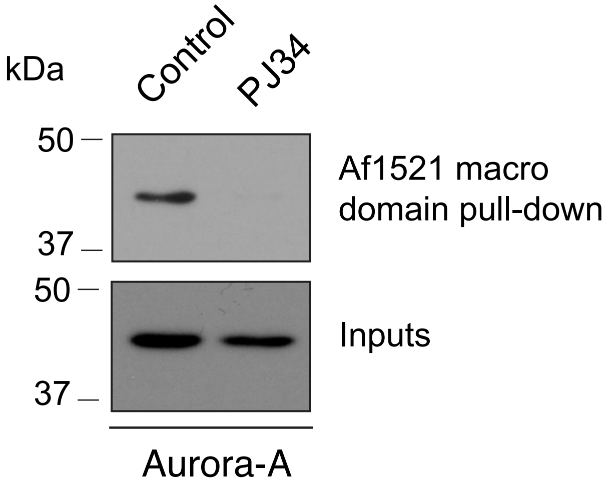

Figure S4
MARylation levels of Aurora A were analyzed using the Af1521 macro domain–based pull-down assay on total cell lysates obtained from synchronized HeLa cells harvested at 8 hours after thymidine release, left untreated (control) or treated with the PARP inhibitor PJ34 (50 μM).

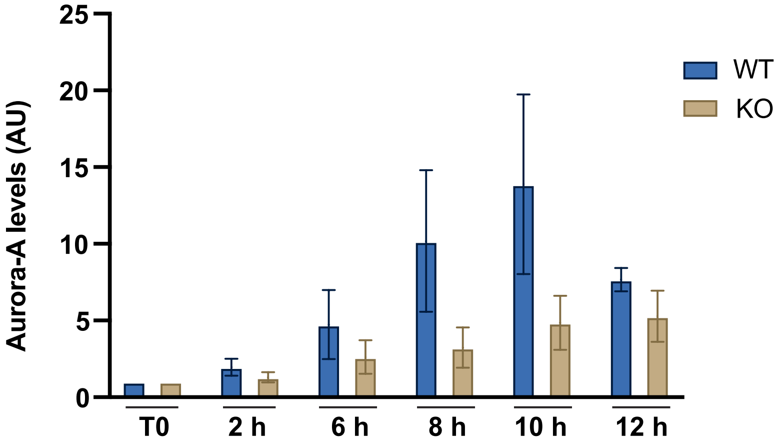

Figure S5
Quantification of Aurora-A protein levels during cell cycle progression, in wild-type and PARP10 KO HeLa cells.

Figure 1a

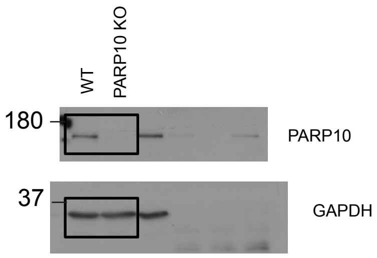

Figure 2a

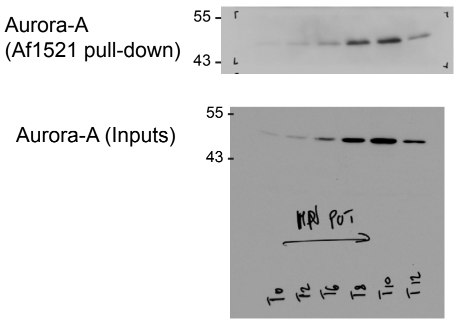

Figure 2b

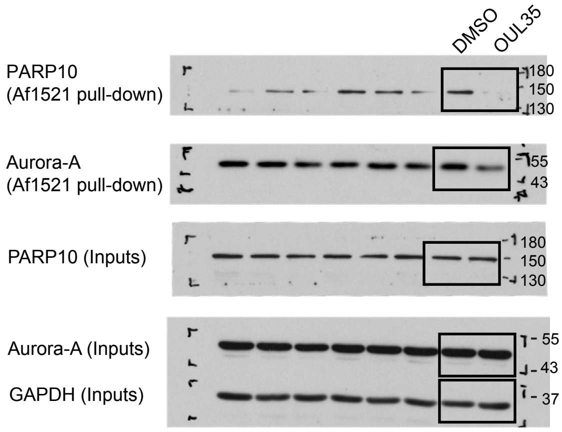

Figure 2c

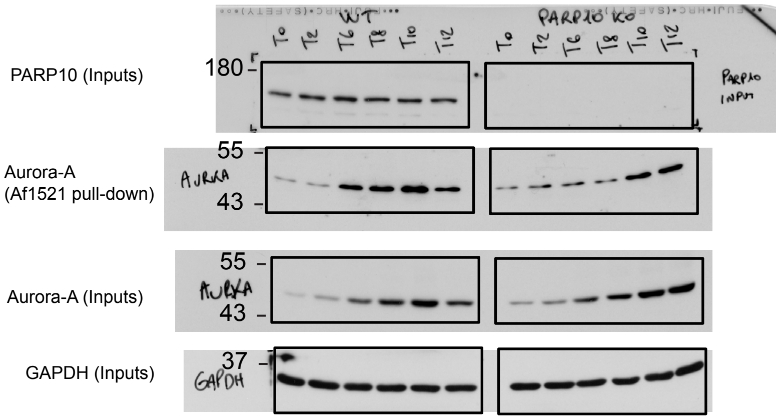

Figure 2e

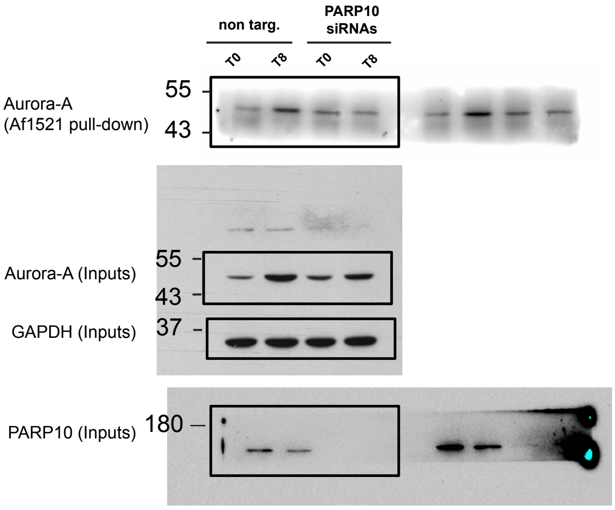

Figure 3b

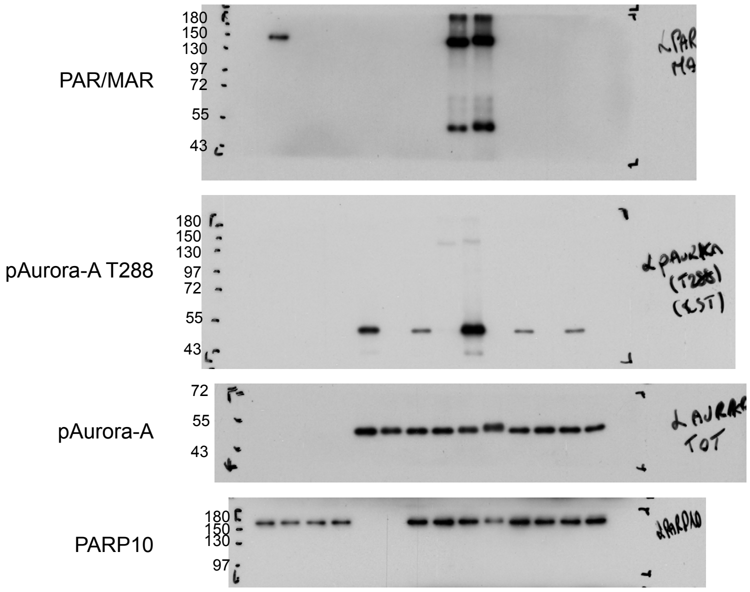

Figure S6

Original blots from all performed Western blots.
